# Dominant Loss Mechanisms of Whispering Gallery Mode RF-MEMS Resonators with Wide Frequency Coverage

**DOI:** 10.3390/s20247017

**Published:** 2020-12-08

**Authors:** Zeji Chen, Qianqian Jia, Wenli Liu, Quan Yuan, Yinfang Zhu, Jinling Yang, Fuhua Yang

**Affiliations:** 1 Institute of Semiconductors, Chinese Academy of Sciences, Beijing 100083, China; chenzeji@semi.ac.cn (Z.C.); jiaqianqian@semi.ac.cn (Q.J.); liuwenli@semi.ac.cn (W.L.); yuanquan@semi.ac.cn (Q.Y.); yfzhu@semi.ac.cn (Y.Z.); fhyang@semi.ac.cn (F.Y.); 2 Center of Materials Science and Optoelectronics Engineering, University of Chinese Academy of Sciences, Beijing 100049, China; 3 State Key Laboratory of Transducer Technology, Shanghai 200050, China

**Keywords:** loss mechanism, MEMS resonator, multi-frequency, quality factor, whispering gallery mode

## Abstract

This work investigates the dominant energy dissipations of the multi-frequency whispering gallery mode (WGM) resonators to provide an insight into the loss mechanisms of the devices. An extensive theory for each loss source was established and experimentally testified. The squeezed film damping (SFD) is a major loss for all the WGMs at atmosphere, which is distinguished from traditional bulk acoustic wave (BAW) resonators where the high-order modes suffer less from the air damping. In vacuum, the SFD is negligible, and the frequency-dependent Akhiezer damping (AKE) has significant effects on different order modes. For low-order WGMs, the AKE is limited, and the anchor loss behaves as the dominant loss. For high-order modes with an extended nodal region, the anchor loss is reduced, and the AKE determines the *Q* values. Substantial *Q* enhancements over four times and an excellent *f* × *Q* product up to 6.36 × 10^13^ at 7 K were achieved.

## 1. Introduction

Micro-electro-mechanical system (MEMS) resonators with small occupation, IC compatibility, and lower power consumption have emerged as a key enabling solution to constitute advanced RF-front transceivers for future wireless communications [1,2,3,4,5]. The bulk acoustic wave (BAW) resonators are extremely attractive for their high stiffness and low energy dissipation [6]. Based on high-performance BAW resonators with promising *f* × *Q* products [7,8,9,10], many outperforming RF components have been demonstrated, such as MEMS oscillators with low phase noise [11,12,13] and RF channel-select filters with ultra-narrow passband [14,15].

Multi-frequency BAW resonators are highly desired for high-end applications such as multi-clock oscillators and multi-band filters. For some widely-used BAW resonators, it is hard to maintain high *f* × *Q* products in high-order modes as the *Q* values degrade sharply due to the severer anchor loss. For example, in contour mode resonators, the *Q* of high-order modes was deteriorated by the increasing displacement at the stem-supporting region [16]. In width extensional mode resonators, the *Q* values in even modes suffer from severe energy loss at the tether attachment point [10]. However, the loss mechanisms of various resonators are different [17]. In some devices, like Lamé mode resonators [18], and composite thin-film piezoelectric-on-substrate (TPoS) resonators vibrating in extensional modes [19], high *Q* values as well as promising *f* × *Q* products have been achieved in high-order modes. Nevertheless, the reported BAW resonators are still operating at single frequencies. The whispering gallery modes (WGMs) with extended nodal region in high-order modes are apt to achieve higher *f* × *Q* products owing to the reduced anchor loss, which is superior to contour or extensional modes. In addition, the standing waves formed at the disk periphery are beneficial for setting multiple electrodes to implement multi-mode excitations [20]. In [21,22], WGM based switchable disk resonators have been demonstrated. Distinguished from traditional single-frequency BAW resonators, multiple modes can be simultaneously excited to achieve wide frequency coverages, and the *Q* values retain over 10^4^ for each mode at atmosphere. To better understand their *Q* limitations, it is significant to study the distinct loss mechanisms of such devices and explore their potential applications.

The reported work mainly focuses on one or several loss sources on certain order WGMs. The anchor loss of WGM resonators has been qualitatively studied in [23,24], which tends to be negligible with increasing mode order, while the dominant dissipations of the high-order modes have not been clarified. The effects of thermoelastic damping (TED) and phonon-phonon interaction damping (PPID) on WGM resonators were analyzed in [25]. It was theoretically indicated that PPID is more significant than TED and determines the upper limit of the *f* × *Q* product. In [26], the anchor loss, TED, and PPID were compared for the 2nd WGM resonator with different supporting configurations at 5 K. It was reported that the anchor loss could still remain dominated. However, for multi-mode WGM resonators with wide frequency coverages, the dominant loss mechanisms of each mode can be different and have not been reported yet; thus, the relevant loss sources should be fully taken into account. In the previous work [22], the energy loss sources for the multi-mode WGM resonators have been preliminarily investigated in different experimental conditions; however, the theoretical models were not built up, although the theoretical analyses are essential for quantitatively evaluating each loss mechanism. 

In this work, extensive theoretical models were established and experimentally verified. With promising agreement between the theoretical analysis and experimental results, the main loss sources in different working conditions were clarified. For the first time, the distinct temperature dependences of *Q* for various order modes were explicitly presented and the limiting loss mechanisms at the cryogenic temperatures could be unveiled. Comprehensive analysis about the energy dissipation of the devices was provided.

## 2. Design and Fabrication

**Figure 1:**
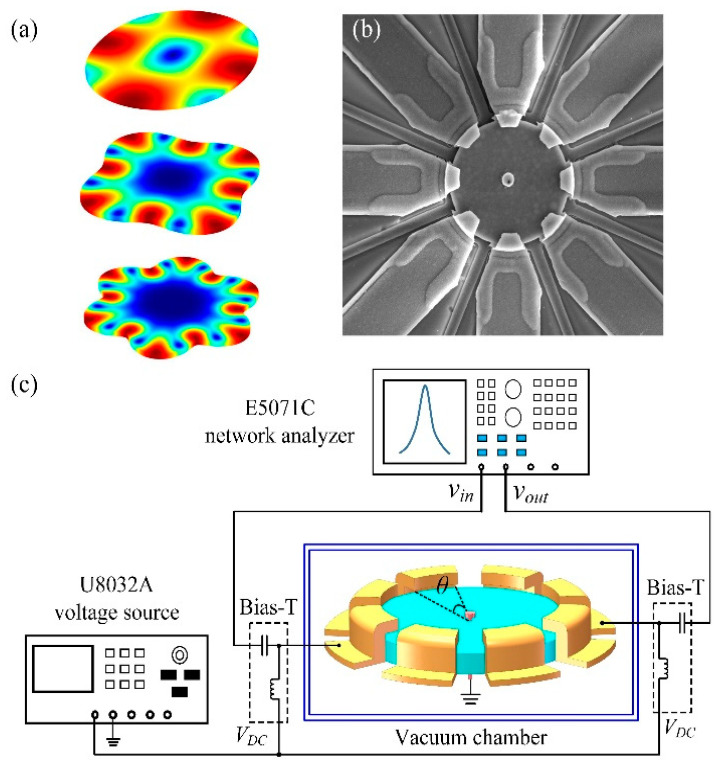
The mode shapes of the 2nd, 4th, and 6th WGMs (**a**); the scanning electron microscope (SEM) photograph of the fabricated resonator (**b**); and the measurement setup of the WGM resonators (**c**).

The disk resonator is surrounded by eight uniformly distributed electrodes at the circumference. Utilizing the one-pair of electrode configuration, i.e., two opposite electrodes, the 2nd to 7th WGMs can be simultaneously excited [22]. The supporting stem is located at the center of the disk, corresponding to the nodal region of the disk; thus, the anchor loss can be minimized [21]. The mode shapes are given in Figure 1a. The resonance frequencies can be calculated by solving the following equations [27]:

(1) $$\left[\psi \left(\frac{\zeta }{\xi }\right)-n-q\right]\left[\psi \left(\zeta \right)-n-q\right]={\left(n\cdot q-n\right)}^{2},$$

(2) $$\psi \left(x\right)=\frac{xJ_{n-1}\left(x\right)}{J_{n}\left(x\right)},\;q=\frac{\zeta^{2}}{\left(2n^{2}-2\right)},$$

(3) $$\zeta =2\pi f_{n}R\sqrt{\frac{\rho \left(2\sigma +2\right)}{E}},\;\xi =\sqrt{\frac{2}{1-\sigma }},$$

where *n* is the mode order; *J_n_* is the Bessel function of the first kind; *f_n_* refers to the resonance frequency of the *nth* mode; *R* denotes the disk radius; and *E*, *ρ*, and *σ* refer to the Young’s modulus, density, and Poisson’s ratio, respectively.

As shown in Figure 1c, with the central disk grounded, an AC signal together with a DC bias voltage were applied to the driving electrode using the bias-T; meanwhile, another DC bias voltage was applied to the sensing electrode to extract the motional current. The span angle of the electrode *θ* is critical for multi-mode excitations. According to dependence of the motional current on the span angle for each mode, the optimal angle of an individual mode is *π*/*n*. A small angle cannot provide sufficient electromechanical couplings for low-order modes, while the large one will suppress the high-order modes. Therefore, a traded-off value of 34° was employed [22]. The three-layer self-aligned process was adopted to batch fabricate the 37 μm and 18 μm-radius WGM resonators [28]. The nano-scale spacing gap of 70 nm was made via the thermal oxidation to reduce the impedance. The low pressure chemical vapor deposition low pressure chemical vapor deposition (LPCVD) device layer with nearly zero stress yielded a higher reliability and reduced energy dissipation. The fabricated resonator is shown as Figure 1b.

## 3. Theoretical Analysis

The total *Q* of the MEMS resonators can be expressed as:

(4) $$\frac{1}{Q}=\sum \frac{1}{Q_{i}},$$

where *i* denotes one type of loss mechanism, and the total *Q* is dominated by the lowest *Q_i_*. The most relevant energy dissipation sources of RF-MEMS resonators are: anchor loss, air damping, TED, and PPID [29]. For a comprehensive analysis, each loss source should be individually modeled.

### 3.1. Anchor Loss

**Figure 2:**
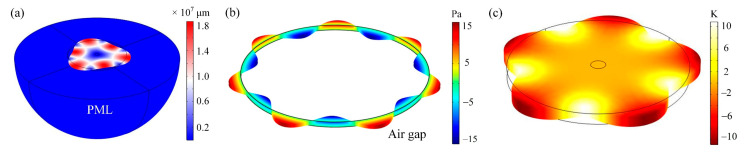
The simulated displacement distribution of the WGM resonator as well as the substrate (**a**), the varied pressure profile of the air gap (**b**), and the temperature distribution (**c**).

The anchor loss is associated with the acoustic waves radiating away from the resonator through the supporting stem and propagating into the substrate. The energy is thus dissipated. As shown in Figure 2a, the disk is suspended by the anchor stem. The bottom end of the stem is attached to the substrate. The semi-infinite substrate is modeled as the semicircle. The perfectly match layers (PML) are applied at the edge of the substrate to absorb the propagating acoustic waves [30]. For multiple WGMs, the thickness of the PML is set as the corresponding resonance wavelength of each mode. The Comsol Multiphysics was used for simulation. Applying the eigenfrequency analysis to the established model, a complex-valued result can be attained, which consists of a real part referring to the resonance frequency and the imaginary part reflecting the exponential damping of the displacement field. Figure 2a shows the displacement distribution of the 3rd WGM. The *Q_anchor_* can be calculated as [31]:

(5) Qanchor=Re(fn)2Im(fn).

### 3.2. Squeezed-Film Damping

During the vibration of MEMS resonators, the air is moved in and out of the nano-scale spacing gap alternatively. A varied pressure field is generated, which induces a significant force on the resonator sidewall against the vibration; a portion of energy is thus dissipated. This effect is referred to as the squeezed-film damping (SFD). To evaluate the *Q_SFD_*, the total energy stored in the device as well as the varied pressure induced energy dissipation should be determined. The stored energy can be calculated using the following equation [32]:

(6) $$E_{total}=\frac{1}{2}\rho h\int_{0}^{2\pi }\int_{0}^{R}{\left[\omega_{n}X\left(r,\theta \right)\right]}^{2}rdrd\theta ,$$

where *h* is the thickness of the disk, *ω_n_* is the angular resonance frequency of the *n*th mode, and *X*(*r*,*θ*) is the displacement of an arbitrary point in the disk, which can be expressed as [22]:

(7) $$X(r,\theta )=Q_{n}\frac{V_{P}V_{i}\varepsilon_{0}R}{\rho \omega_{n}^{2}g_{0}{}^{2}}\frac{X_{mode}(r,\theta )\int_{-\frac{\varphi }{2}}^{\frac{\varphi }{2}}X_{\mathrm{mod}e}(r,\theta^{\prime })d\theta^{\prime }}{\int_{0}^{2\pi }\int_{0}^{R}{\left[X_{mode}\left(r^{\prime \prime },\theta^{\prime \prime }\right)\right]}^{2}r^{\prime \prime }dr^{\prime \prime }d\theta^{\prime \prime }},$$

where *V_p_*, *V_i_*, and *Q_n_* are the DC bias voltage, AC voltage amplitude, and the quality factor of the *n*th mode, respectively; *ε*_0_, *φ*, and *g*_0_ denote permittivity, the span angle of electrode, and the spacing gap, respectively; and *X*_mode_ is the normalized mode shape function [32]:

(8) $$X_{\mathrm{mode}}=\left[\frac{d}{dr}J_{n}(k_{n}\frac{r}{R})+\frac{n}{r}\frac{N_{n}}{M_{n}}J_{n}(h_{n}\frac{r}{R})\right]\cos n\theta ,$$

where *k_n_* and *h_n_* are the frequency associated parameters, which can be expressed as:

(9) $$\begin{array}{l} k_{n}=\omega_{n}\frac{R}{\sqrt{\frac{E}{\rho \left(1-\upsilon^{2}\right)}}},\\ h_{n}=\omega_{n}\frac{R}{\sqrt{\frac{E}{2\rho \left(1+\upsilon \right)}}}. \end{array}$$

The relationship between parameters *M_n_* and *N_n_* is [20]:

(10) $$\frac{N_{n}}{M_{n}}=\frac{J_{n}\left(k_{n}\right)}{J_{n}\left(h_{n}\right)}\frac{2\frac{k_{n}J_{n-1}\left(k_{n}\right)}{J_{n}\left(k_{n}\right)}+h_{n}^{2}-2n\left(n+1\right)}{\left[\frac{h_{n}J_{n-1}\left(h_{n}\right)}{J_{n}\left(h_{n}\right)}-\left(n+1\right)\right]\cdot 2n}.$$

Then, the energy dissipation due to the SFD should be extracted. The varied pressure inside the gap is described through the Reynold’s equation [33]:

(11) $$P_{a}\nabla^{2}(\delta p)-\frac{12\eta_{eff}}{g_{0}^{2}}\frac{\partial (\delta p)}{\partial t}=\frac{12\eta_{eff}}{g_{0}^{3}}\frac{dX(R,\theta ,t)}{dt},$$

where *δp*, *P_a_*, and *X*(*R*,*θ*,*t*) are the variation pressure, ambient pressure, and time-harmonic displacement at the circumference of the disk, respectively, and *η_eff_* is the effective viscosity, which can be expressed as [34]:

(12) $$\eta_{eff}=\frac{\eta_{0}}{1+9.639K_{n}{}^{1.159}},$$

where *η*_0_ denotes the viscosity in atmosphere, and *K_n_* is the Knudsen number associated with the ambient pressure:

(13) $$K_{n}=\frac{P_{0}}{P_{a}}\frac{\lambda_{0}}{g_{0}},$$

where *λ*_0_ refers to the mean free path at normal atmospheric pressure *P*_0_.

The varied pressure field of the 7th WGM is given in Figure 2b. Furthermore, with the varied pressure field and the displacements at the circumference numerically solved, the energy dissipation per period *W_loss_* can be extracted according to the following equation:

(14) $$W_{loss}=\int_{0}^{T_{n}}\omega_{n}\left(\iint_{S}\delta pX(R,\theta ,t)dS\right)dt,$$

where *T_n_* is the vibrational period of the *n*th mode, and *S* refers to the area of the disk sidewall. The *Q_SFD_* can be calculated via:

(15) $$Q_{SFD}=2\pi \frac{W_{total}}{W_{loss}}.$$

### 3.3. Thermal Elastic Damping

The TED results from the irreversible heat flow arising from the temperature gradients associated with the differential volumetric changes [35]. For an isotropic thermoelastic solid with arbitrary geometrical shapes, the temperature distribution should be firstly attained via the thermodynamic equation [36,37]:

(16) $$\kappa \nabla^{2}T-C_{v}\frac{\partial T}{\partial t}-\alpha (3\lambda +2\mu )T_{ref}(\frac{\partial }{\partial x}\frac{\partial u}{\partial t}+\frac{\partial }{\partial y}\frac{\partial v}{\partial t}+\frac{\partial }{\partial z}\frac{\partial w}{\partial t})=0,$$

where *C_v_* and *κ* are the volumetric heat capability and thermal conductivity [38], respectively; *u*, *v*, and *w* are the displacements in the *x*, *y*, and *z* axes; *λ* and *μ* refer to the elastic Lamé parameters; *T_ref_* and *T* denote the ambient and distributed temperature, respectively; and *α* is the thermal expansion coefficient [39]. Then, the temperature distribution should be incorporated into the mechanical domain. Taking the thermal-mechanical equation in the *x* direction as an example, the expression takes the form as:

(17) $$\rho \frac{\partial^{2}u}{\partial t^{2}}=µ(\frac{\partial^{2}u}{\partial x^{2}}+\frac{\partial^{2}u}{\partial y^{2}}+\frac{\partial^{2}u}{\partial z^{2}})+(\lambda +µ)(\frac{\partial^{2}u}{\partial x^{2}}+\frac{\partial^{2}v}{\partial x\partial y}+\frac{\partial^{2}w}{\partial x\partial z})-\alpha (3\lambda +2µ)\frac{\partial T}{\partial x}.$$

Based on the coupling equations, with the reference temperature *T*_0_ given, similar to (2), a complex *ω* can be obtained and the *Q* can be extracted. The temperature distribution of the 5th WGM is shown in Figure 2c.

### 3.4. Phonon-Phonon Interaction Damping

The phonon-phonon interaction damping (PPID) arises from the scattering of the acoustic phonons. When the sound wavelength (*λ_s_*) is significantly larger than the phonon mean free path, i.e., *fτ_ph_* << 1, where *τ_ph_* is the thermal relaxation time, the acoustic wave is assumed to interact with the whole ensemble of thermal phonons. This process is referred as the Akhiezer regime (AKE), which is valid for resonance frequencies within hundreds of MHz. On the contrary, if *λ_s_* is less than the mean free path, the acoustic quanta will interact with the lattice phonons individually, and the process is referred as the Landau Rumer regime (L-R) [38,40], which is valid for very high frequencies exceeding GHz. The WGM resonators with frequencies ranging from 53 to 362 MHz are vibrating in the AKE regime. The *Q_AKE_* takes the form as [41]:

(18) $$Q_{AKE}=\frac{\rho v_{a}{}^{2}}{C_{v}T\gamma_{eff}{}^{2}}\frac{1+{\left(\omega_{n}\tau_{ph}\right)}^{2}}{\omega_{n}\tau_{ph}},$$

where *v_a_* and *γ_eff_* are the averaged sound velocity and effective Grüneisen parameter, respectively. The *γ_eff_* characterizes the phonon frequency variations when the crystal volume is changed [42], which is dependent on the mode shape, material properties, crystal orientation, wave propagation direction, and so on [43]. In this work, an averaged value of 0.51 was employed [44]. The relaxation time *τ_ph_* takes the form as [41]:

(19) $$\tau_{ph}=\frac{3\kappa }{C_{v}V_{D}^{2}},$$

where *V_D_*, the mean Debye velocity, can be determined by:

(20) $$\frac{1}{V_{D}{}^{3}}=\frac{1}{V_{l}{}^{3}}+\frac{2}{V_{t}{}^{3}},$$

where *V_l_* and *V_t_* are the longitudinal and transverse velocities, respectively [44]. It should be noted that in the Akhiezer regime, *Q_AKE_* is proportional to *f*^−1^ for longitudinal waves, and proportional to *f*^−0.75^ for transverse waves [38]. For WGMs with both the P- and S-wave contributed, the *Q_AKE_* could have a complicated frequency-dependence of *f^-r^* with *r* between 0.75 and 1. As can be seen, *Q_AKE_* still decreases monotonically with the frequency. In addition, thermal conductivity *κ* varies with the ambient temperature and reduces at lower temperatures [45]. According to Equations (18) and (19), at cryogenic temperatures, the AKE will be effectively suppressed and the AKE-dominated vibrating modes will gain substantial *Q* enhancements. To sum up, despite the relatively complicated dependence of *Q_AKE_* on the temperature and frequency, (18) is sufficient to evaluate *Q_AKE_* values at different temperatures and provides a deep understanding about the distinct AKE effects on multi-mode resonators. The material parameters for different *Q* calculations are summarized in Table 1.

## 4. Results and Discussions

A series of measurements were accomplished to characterize the loss mechanisms of WGM resonators in different conditions. The measurement setup is shown in Figure 1c.

### 4.1. Resonator Performance in Air

**Figure 3:**
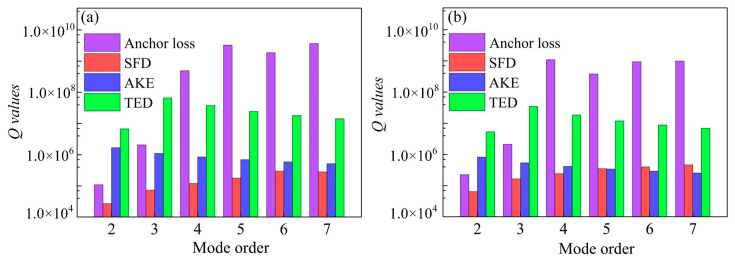
Comparison between the *Q_anchor_* and *Q_SFD_* values of each WGM for the 37 μm (**a**) and 18 μm-radius resonators (**b**).

A group of resonators were firstly tested at atmosphere; the measured *Q* values are summarized in Table 2. For 37 and 18 μm-radius resonators, the calculated *Q* values using Equations (1)–(3) match well with the measured ones. The discrepancies can be attributed to the slight differences between the real and theoretical parameter values, process tolerances, and so on. The *Q* values of all the modes up to the 7th WGM maintain around 10^4^, indicating that the anchor loss could not be a major loss source for the high-order modes. The *Q_anchor_* values of each mode for both 37 and 18 μm-radius devices were simulated based on the described model in Section 3, Part 1. As shown in Figure 3, the *Q_anchor_* of the 2nd to 4th modes rises up from 10^5^ to 10^9^ as the nodal region is expanding towards the circumference, and then it retains around 10^9^ for the 4th to 7th modes. These results infer that the anchor loss is negligible in high-order WGMs. 

It should be noted that for WGMs, along the radial direction, the normalized motion at a given point tends to decrease with the increasing mode order [21]; thus, the *Q_anchor_* values are expected to rise with the mode order. However, the simulated *Q* values do not increase monotonously. Additionally, supposing that the energy within the stem region is all dissipated, the ratio of the leakage energy to the total energy takes the form as:

(21) $$\frac{E_{stem}}{E_{total}}=\frac{\frac{1}{2}\rho h\int_{0}^{2\pi }\int_{0}^{R_{s}}{\left[\omega_{n}X\left(r,\theta \right)\right]}^{2}rdrd\theta }{\frac{1}{2}\rho h\int_{0}^{2\pi }\int_{0}^{R}{\left[\omega_{n}X\left(r,\theta \right)\right]}^{2}rdrd\theta }=\frac{\int_{0}^{2\pi }\int_{0}^{R_{s}}{\left[\omega_{n}X_{\mathrm{mod}e}\left(r,\theta \right)\right]}^{2}rdrd\theta }{\int_{0}^{2\pi }\int_{0}^{R}{\left[\omega_{n}X_{\mathrm{mod}e}\left(r,\theta \right)\right]}^{2}rdrd\theta },$$

where *R_s_* refers to the stem radius. Assuming $\beta =\frac{r}{R}$ and substituting Equation (8) into (21) yields:

(22) $$\frac{E_{stem}}{E_{total}}=\frac{\int_{0}^{2\pi }\cos n\theta d\theta \int_{0}^{\frac{R_{s}}{R}}{\left[\frac{d}{d\beta }J_{n}(k_{n}\beta )+\frac{n}{\beta }\frac{N_{n}}{M_{n}}J_{n}(h_{n}\beta )\right]}^{2}\beta d\beta }{\int_{0}^{2\pi }\cos n\theta d\theta \int_{0}^{1}{\left[\frac{d}{d\beta }J_{n}(k_{n}\beta )+\frac{n}{\beta }\frac{N_{n}}{M_{n}}J_{n}(h_{n}\beta )\right]}^{2}\beta d\beta }.$$

The stem radii of the 37 and 18 μm-radius resonators are 3 μm and 1.5 μm, respectively. Pursuant to (22), with similar stem to disk radii ratios, the *Q_anchor_* values of each mode should be very close; however, the simulations exhibit clear difference. The discrepancies can be caused by the supporting stem, which is modeled as a longitudinal rod subjected to the time-harmonic vibrations with the resonance frequency *f*_n_. The height and radius of the supporting stem affect the wave propagations from the disk to the substrate, leading to the *Q_anchor_* values different from the expectation [46].

### 4.2. Resonator Performance in Vacuum

For WGM resonators with a nanoscale spacing gap, the squeezed film damping could be significant. To provide a clear comparison between the SFD and anchor loss, the *Q_SFD_* values of the 18 and 37 μm-radius resonators were calculated and plotted in Figure 3 to compare with the *Q_anchor_* values. As can be seen, the *Q_SFD_* values are much smaller than the *Q_anchor_* values even in the 2nd mode with severe anchor loss. Clearly, there is more SFD than the anchor loss, especially in the high-order WGMs. Substantial *Q* enhancements are expected for all the WGMs in vacuum.

**Figure 4:**
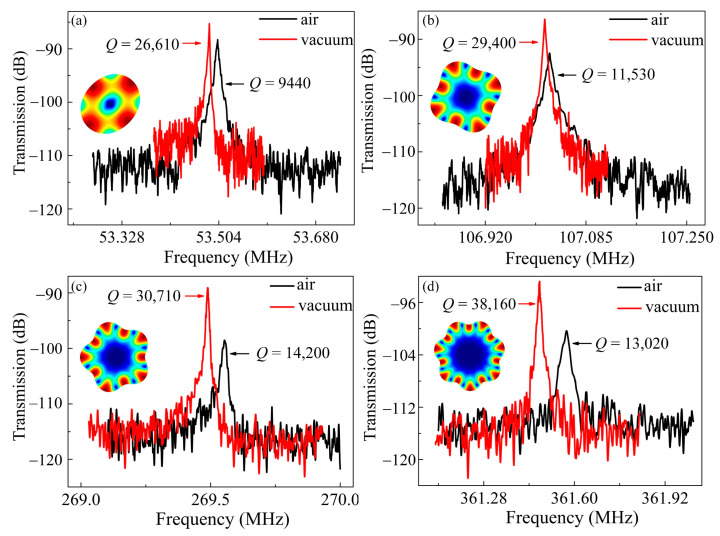
The frequency responses in air and vacuum for the 2nd and 4th WGMs of the 37 μm-radius resonator (**a**,**b**) as well as for the 5th and 7th WGMs of the 18 μm-radius resonator (**c**,**d**).

The devices were subsequently tested in vacuum. A Lakeshore CRX-4K probe station was employed to provide a high vacuum of 8 × 10^−5^ bar. For different WGMs, *Q* enhancements exceeding 2 times were achieved. As shown in Figure 4a,b, for the 2nd and 4th WGMs of the 37 μm-radius resonator, the *Q* improves from 9440 to 26,610, and from 11,530 to 29,400, respectively. The similar tendency can be obtained for the 18 μm-radius counterparts. Figure 4c,d show that for 5th and 7th WGMs, the *Q* improves from 14,200 to 30,710, and from 13,020 to 38,160, respectively. An outperforming *f* × *Q* product up to 1.38 × 10^13^ was implemented. The substantial *Q* enhancements of all the WGMs infer that the SFD is a major loss source even for high-order modes. By contrast, the traditional high-order BAW modes are insensitive to the SFD due to the increased stiffness [16,47]. The resonance frequency shift could be attributed to the effect of hydrostatic pressure. The bulk elastic modulus goes down with the decreasing pressure [48], which leads to a reduced frequency.

Despite the fact that the anchor loss and SFD are negligible in vacuum, the *Q* values of the high-order WGMs are not as high as expected. There could be some other loss sources that limit the *Q* enhancements of high-order WGMs. The TED and AKE, which could be more significant at higher frequencies, should be taken into account.

### 4.3. Resonator Performance at Cryogenic Temperatures

The TED and AKE can be effectively suppressed at cryogenic temperatures. The high-order WGMs, which could be dominated by these two mechanisms, would exhibit higher *Q* enhancements at low temperatures. For both two types of resonators, the *Q* values were measured with the temperatures ranging from 7 K to 293 K, and the step was set as 16 K.

**Figure 5:**
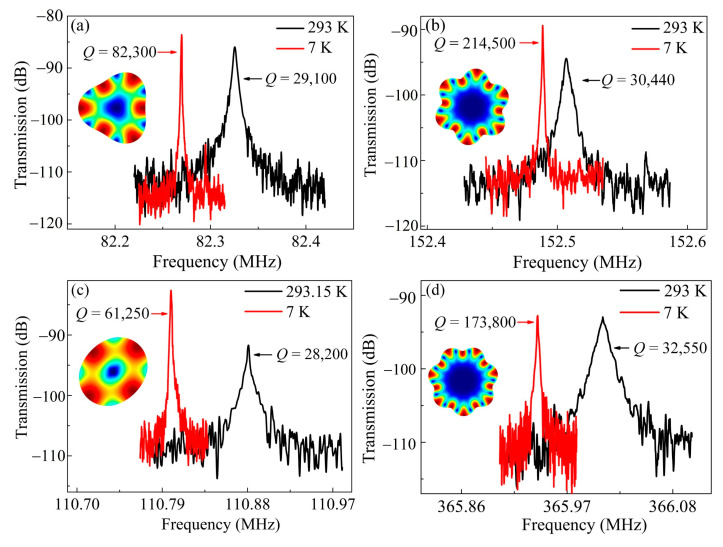
The frequency responses at the room and cryogenic temperatures of the 3rd and 6th WGMs for the 37 μm-radius resonator (**a**,**b**), and the 2nd and 7th WGMs for the 18 μm-radius resonator (**c**,**d**).

The measured frequency responses for the 37 and 18 μm-radius resonators at 7 K are given in Figure 5. As can be seen, the *Q* of the 3rd mode for the 37 μm-radius resonator at 7 K is 82,300, and relative to the *Q* of 29,100 at room temperature, the enhancement is only 2.83 times (Figure 5a). By contrast, the *Q* in the 6th mode boosted from 30,440 to 214,500 at 7 K (Figure 5b), which achieves a promising enhancement of 7.05 times. The 18 μm-radius counterparts exhibit the similar tendency. As shown in Figure 5c,d, the *Q* values of the 2nd and 7th WGMs at room temperature were 28,200 and 32,550, and were raised up to 61,250 and 173,800 at 7 K, corresponding to the enhancements of 2.17 and 5.34 times, respectively. Besides, an outperforming *f* × *Q* product up to 6.36 × 10^13^ of the 7th WGM was implemented at 7 K. The frequency shifts are determined by the frequency-temperature (*f*-*T*) coefficients at cryogenic temperatures. For highly p-doped shear mode resonators, the *f*-*T* curve is a parabola over a wide temperature range, wherein the frequency goes down with the decreasing temperature in the low temperature range [49]. For highly p-doped WGM resonators with mainly shear deformations, the *f*-*T* coefficient tends to be positive at cryogenic temperatures, which gives rise to the frequency reduction.

As expected, the *Q* enhancements of high-order WGMs benefit more from the temperature reduction. Given that both AKE and TED dramatically decrease at low temperatures, these two sources are difficult to be distinguished experimentally, but can be separated theoretically. The *Q_AKE_* and *Q_TED_* values of the 37 and 18 μm-radius resonators were calculated using the established models and also plotted in Figure 3 to provide a clear comparison. As can be seen, the *Q_AKE_* decreases monotonically with the frequency and tends to approach the SFD in high-order modes. Therefore, the AKE contributes to a substantial portion of energy dissipation for high-order WGMs at room temperature, which leads to their limited *Q* improvements in vacuum. As for TED, it is identified as a negligible loss source in BAW modes, especially in volume conserved WGMs [6], which is verified via the calculated results as shown in Figure 3. It is worth mentioning that both the AKE and TED are not simply inversely proportional to the temperature [39] since the thermal expansion coefficient *α* in (16) and thermal conductivity *κ* in (19) are temperature-dependent variations. However, as demonstrated in [26], the *Q_AKE_* retains much lower than the *Q_TED_* from 5 K to 293 K; hence, the TED can be ruled out as a major loss source for the devices.

It should be noted that with AKE suppressed at lower temperatures, the other loss mechanisms could override it and become dominated. Thus, there could be a turnover temperature in the *Q-T* curve of an individual WGM. Between the turnover and room temperatures, the *Q* would decrease with the increasing temperature, which could take a form as an approximate negative power function. Given that the high-order modes have more striking *Q* enhancements, their *Q* dependences on the temperature are expected to be stronger. On the other hand, when the temperature is lower than the turnover point, the *Q* would be insensitive to the temperature. For high-order modes, since the AKE contributes more energy dissipation, it would be dominated within a wider temperature range. Therefore, the turnover temperatures of the high-order WGMs are supposed to be lower than the low-order ones.

**Figure 6:**
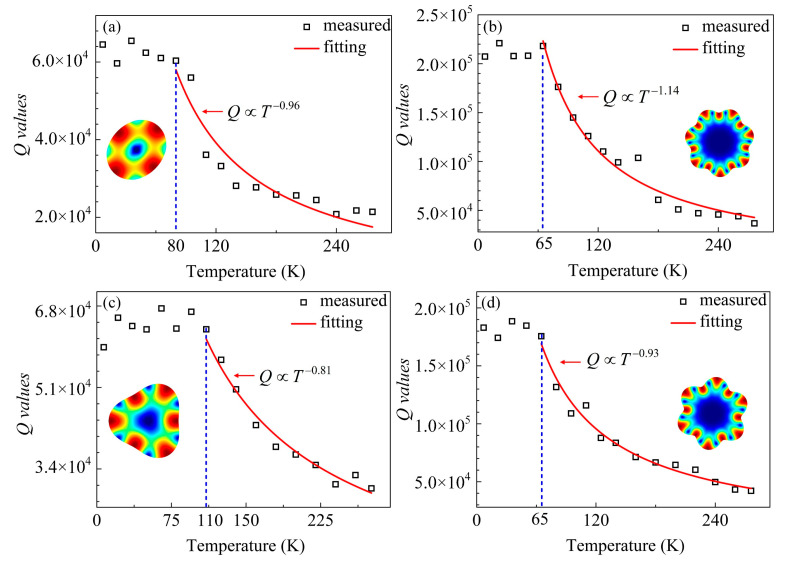
The *Q* values vs. temperature of the 2nd and 7th modes for the 37 μm-radius resonator (**a**,**b**), and of the 3rd and 6th modes for the 18 μm-radius resonator (**c**,**d**).

The *Q* values vs. the temperature are plotted in Figure 6. As can be seen from Figure 6a,b, for the 37 μm-radius resonator, the turnover temperature of the 2nd and 7th modes are 80 K and 65 K, respectively, indicating that the AKE plays a more important role in the high-order modes. The *Q*-*T* dependences were quantitively evaluated using the allometric fitting method with the temperature between the turnover points and 293 K. The *Q* dependence of *T***^−^**^1.14^ for the 7th WGM was stronger than that of *T***^−^**^0.96^ for the 2nd WGM. Similarly, for the 18 μm-radius counterparts, as shown in Figure 6c,d, the turnover temperatures of the 3rd and 6th WGMs occurred at 110 K and 65 K, corresponding to the *Q* dependences of *T***^−^**^0.81^ and *T***^−^**^0.93^, respectively. For both two types of resonators, with temperatures below the turnover points, the *Q* values are insusceptible to the temperature, consistent with the tendency that the AKE can be overridden by other loss sources at lower temperatures. Furthermore, as illustrated in Figure 3, for the low-order modes, the temperature-independent anchor loss becomes the limiting mechanism when the temperature drops below the turnover point. As for the high-order modes, the anchor loss is negligible, the fabrication process induced damage and defects might be the main loss source [50,51].

Combining with the theoretical analysis and the experimental results, the distinct loss mechanisms of WGM resonators can be inferred. The SFD is a major loss source for WGM resonators even with high-order modes and high stiffness, while the AKE effect in high-order WGMs is nonnegligible. In vacuum, the anchor loss and AKE become the *Q* limitations for low-order and high-order modes, respectively, which have been testified with the cryogenic temperature tests.

Pursuant to the dominant loss mechanisms of various vibration modes, strategies can be taken to further improve the *Q* values. For low-order modes, the anchor loss can be reduced by optimizing the stem design [52] or introducing acoustic impedance discontinuity in the energy transmission path. Fabricating disks and stems with different materials is an effective routing [53]. As for high-order modes, optimizing the fabrication process could reduce the underlying process-induced energy dissipation [54].

### 4.4. Comparison between Theoretical and Experimental Results

**Figure 7:**
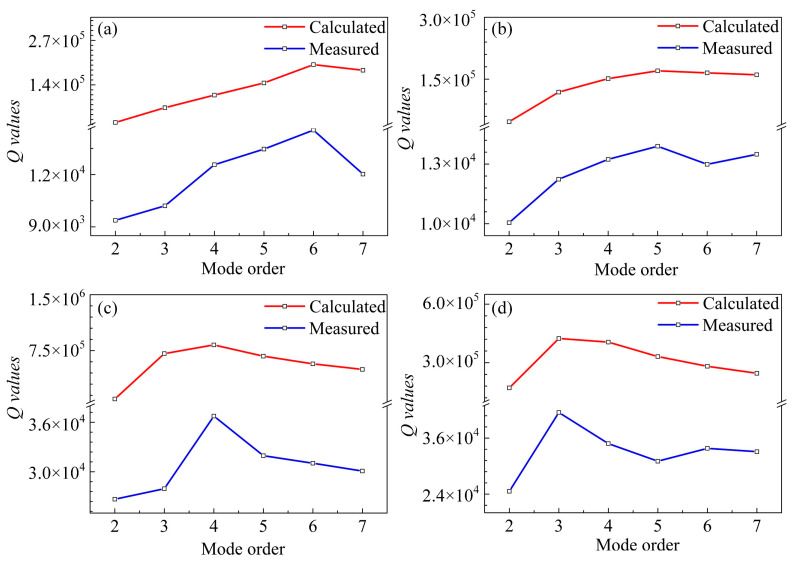
The comparison between the theoretical and measured *Q* values for the 37 and 18 μm-radius resonators in air (**a**,**b**) and vacuum (**c**,**d**).

With the *Q* values of individual loss sources are explicitly expressed, the overall values in different conditions can be evaluated using (4). To further verify the efficiency of the proposed theory, the theoretical values are compared with the measured results. As shown in Figure 7, for both 37 and 18 μm-radius resonators in air and vacuum, the calculated and measured tendencies are consistent. However, the calculated values are far beyond the experimental ones. Firstly, the simulations were carried out based on the ideal models, while the actual *Q* values can be affected by the fabrication process tolerances, parasitic effects, and so on. In addition, the one-pair of electrode configuration with the traded-off electrode angle was employed for multi-mode excitations. When propagating in the whole disk, the vibrations driven with one electrode are more prone to decay due to various loss sources and defects in the device. Besides, the limited driving force results in the sensing signals more susceptible to the distortions caused by feedthroughs. What is more, some other losses, like electrical damping, process induced damage, and surface loss, were not involved. These losses could contribute to the energy dissipation. According to (4), the calculated *Q* values could be overestimated due to the absence of such loss sources.

The theoretical and experimental *Q* enhancements for the 37 and 18 μm-radius resonators are summarized in Table 3. As can be seen, in vacuum, the calculated enhancements are significant for all the WGMs. At cryogenic temperature, the enhancements in the 2nd and 3rd modes are limited, while those in higher-order modes are substantial. The calculations are in accordance with the measured results. However, the calculated *Q* enhancements exhibit more significant differences among various mode orders, especially at 7 K. As indicated by Figure 6, the divergence is attributed to the losses that surpass the AKE at lower temperatures, which are not taken into account in the theoretical model. Thus, the calculated *Q* enhancements are actually overestimated. Referring to these underlying losses as *Q_others_*, according to (4), it can be accessed via:

(23) $$Q_{others}=\frac{1}{\frac{1}{Q_{measured}}-\frac{1}{Q_{calculated}}}.$$

For both two types of devices, the *Q_others_* of all the WGMs at room and cryogenic temperatures are around 10^4^ and 10^5^, respectively. Therefore, the overall effects of the underlying losses could depend on the temperature but not strongly on the resonator dimensions.

## 5. Conclusions

In this work, the distinct loss mechanisms of multi-mode WGM resonators with wide frequency coverages are deeply investigated.

A comprehensive theory was established and matched well with the experimental results. The dominant loss mechanisms can be clearly determined.

The SFD is verified to be a primary loss source for all the WGMs in air. In vacuum, the distinct temperature-dependences of various order modes were clarified. Anchor loss and AKE play important roles in the energy dissipations for the low-order and high-order WGMs, respectively.

Helpful guidance was provided. With the vacuum encapsulations, increasing *f* × *Q* products in high-order modes can be implemented to meet the growing demands of future wireless communications.

Above all, this study provides an insight into the loss mechanisms of the multi-mode WGM resonators and makes a substantial step forward their high-end applications.

## Figures and Tables

**Table 1:** The measured *Q* values of the 37 and 18 μm-radius resonators vibrating in the atmosphere.

Parameter	Value	Parameter	Value
*E*	160 GPa	*μ*	65.57 GPa
*ρ*	2320 kg/m^3^	*α*	2.6 ppm/K
*σ*	0.22	*v_a_*	8305 m/s
*C_v_*	1.65 × 10^6^ J/(m^3^·K)	*V_D_*	5717 m/s
*κ*	34 W/(m·K)	*τ_ph_*	1.89 ps
*λ*	51.52 GPa	*γ_eff_*	0.51

**Table 2:** The measured *Q* values of the 37 and 18 μm-radius resonators vibrating in the atmosphere.

Mode Order	37 μm			18 μm		
	Freq. (MHz)		*Q*	Freq. (MHz)		*Q*
	Cal.	Meas.		Cal.	Meas.	
2	53.60	53.50	9440	110.17	110.11	10,080
3	82.11	82.70	10,400	168.77	170.46	11,550
4	106.71	107.02	11,530	219.35	221.70	13,060
5	129.74	130.58	13,890	266.70	269.55	14,540
6	152.03	152.51	10,200	312.51	315.95	11,640
7	173.90	175.11	12,450	357.50	361.57	12,800

**Table 3:** Calculated and measured *Q* enhancements for the 37 and 18 μm-radius resonators.

	37 μm-Radius Resonator				18 μm-Radius Resonator			
	$\frac{Q_{vacuum,\;293\;K}}{Q_{air,\;293\;K}}$		$\frac{Q_{vacuum,\;7\;K}}{Q_{vacuum,\;293\;K}}$		$\frac{Q_{vacuum,\;293\;K}}{Q_{air,\;293\;K}}$		$\frac{Q_{vacuum,\;7\;K}}{Q_{vacuum,\;293\;K}}$	
	Cal.	Meas.	Cal.	Meas.	Cal.	Meas.	Cal.	Meas.
2	4.75	2.84	1.07	2.53	3.65	2.45	1.25	2.16
3	10.76	2.73	2.39	2.48	3.57	3.50	3.82	2.33
4	7.93	2.93	36.23	3.51	2.68	2.74	38.51	3.50
5	4.79	2.37	39.13	3.94	1.95	2.34	37.91	4.12
6	2.92	2.13	39.17	7.10	1.70	2.71	39.17	5.53
7	2.77	2.50	39.67	6.22	1.53	2.56	39.44	5.23
